# Antibacterial Effect of Canine Leucocyte Platelet-Rich Plasma (L-PRP) and Canine Platelet-Poor Plasma (PPP) Against Methicillin-Sensitive and Methicillin-Resistant *Staphylococcus pseudintermedius*

**DOI:** 10.3390/vetsci11120670

**Published:** 2024-12-20

**Authors:** Roberta Perego, Gabriele Meroni, Piera Anna Martino, Eva Spada, Luciana Baggiani, Daniela Proverbio

**Affiliations:** 1 Veterinary Transfusion Research Laboratory (REVLab), Department of Veterinary Medicine and Animal Sciences, University of Milan, Via dell’Università 6, 26900 Lodi, Italy; eva.spada@unimi.it (E.S.); luciana.baggiani@unimi.it (L.B.); daniela.proverbio@unimi.it (D.P.); 2 One Health Unit, Department of Biomedical, Surgical, and Dental Sciences, University of Milan, Via Pascal 36, 20133 Milan, Italy; gabriele.meroni@unimi.it

**Keywords:** L-PRP, PPP, *S. pseudintermedius*, dog, antimicrobial effect

## Abstract

*Staphylococcus pseudintermedius* (SP) is a commensal and opportunistic pathogen of skin and mucosal surfaces, isolated from healthy dogs and from canine pyoderma cases. It has recently gained attention due to its increasing antibiotic resistance. Platelet-rich plasma (PRP) is a biological product, obtained through a blood centrifugation process, which has antibacterial properties evidenced by in vitro and in vivo studies conducted in both the human and veterinary field. This in vitro study evaluated the antimicrobial effect of canine non-activated and activated leucocyte-rich PRP (L-PRP) and platelet-poor plasma (PPP) against two strains of SP isolated from dogs with pyoderma: one a multidrug-resistant strain (MDR) and one a non-MDR strain. Twenty healthy un-sedated adult blood donor dogs were enrolled for L-PRP and PPP production via a closed semi-automatic system for veterinary use. The evaluation of antimicrobial effect was performed using the micro-inhibition in broth method, exposing SP strains to 10 L-PRP, 10 activated L-PRP and 10 PPP samples, respectively. Bacterial growth was evaluated using CFU count at three timepoints (immediately after incubation T0, after 1 h T1 and after 2 h T2). L-PRP and PPP had a significant antimicrobial effect at all three timepoints which was similar against both non-MDR and MDR SP strains. Activation appeared to reduce the duration of the antimicrobial effect in L-PRP. More studies are necessary to confirm these preliminary results.

## Simple Summary

*Staphylococcus pseudintermedius* (SP) is a commensal and opportunistic pathogen of canine skin and mucosal surfaces that rapidly gained attention due to its increasing antibiotic resistance. Platelet-rich plasma (PRP) is an autologous biological product, which has antibacterial properties, obtained from blood using a centrifugation process. The aim of this study was evaluating the antimicrobial effect of canine leucocyte-rich PRP (L-PRP) and platelet-poor plasma (PPP) against two field strains of SP isolated from dogs affected by pyoderma: one MDR strain and one non-MDR strain, using the micro-inhibition in broth method. L-PRP and PPP had a similar significant antimicrobial effect against both non-MDR and MDR SP strains. More studies are necessary to confirm these results, considering the rise in MDR and pan-drug-resistant bacteria.

## 1. Introduction

*Staphylococcus pseudintermedius* (SP) is a commensal and opportunistic pathogen frequently isolated from the skin and mucosal surfaces of healthy dogs [1]. Isolation rates vary between 46% and 92% depending on body sites sampled [2], but SP is also frequently isolated from canine ear and wound infections [3,4,5] and is recognized throughout the world as the major cause of canine pyoderma [2]. Over the last years it has rapidly gained attention in both veterinary and human medicine due to its increasing antibiotic resistance—especially the methicillin-resistant (MRSP) clones [6], and the carriage of specific virulence factors (e.g., leukocidin, exfoliative toxins, and enterotoxins) making this pathogen a zoonotic agent [7,8].

Methicillin resistance in SP has developed and spread around the world during the past 20 years; according to Hartantyo and colleagues, 63% of SP strains isolated from sick dogs in Singapore are MRSP [9], and these usually demonstrate multidrug resistance (MDR) to three or more antibiotics commonly employed in veterinary care [6,9,10], although in other countries the percentages of MRSP are lower (e.g., 30% USA, 7% Netherlands and Germany, 17% France, and 32% Italy) [11,12,13,14], Additionally, SP is becoming more frequently isolated in people, particularly following direct contact with dogs [15,16]. MRSP may also spread from a sick to a healthy dog by direct or indirect environmental transmission [17].

MDR bacteria are therefore a significant global public health issue with importance in environmental sciences, food safety, and in human, animal, and plant health [18]. The problem in veterinary medicine is particularly pronounced in specific fields, one of which is dermatology, where the use of antibiotics is widespread and frequently poorly controlled [19,20,21,22]. The treatment of SP infections is therefore now considered a serious challenge in veterinary medicine [23] and, as in the human field, veterinary scientific research is looking for alternative therapeutics that can support or replace the action of antibiotics [8,24,25].

Platelet-rich plasma (PRP) is an autologous biological product obtained from blood using a centrifugation process that produces a plasma fraction with a platelet concentration higher than baseline [26]. The clinical efficacy of PRP depends on its concentration of useful platelets and growth factors, which can start cell activation and related signaling pathways. When activated in vivo or in vitro, platelets produce several growth factors that are crucial to start the healing process at the site of the injury. They can stimulate cell proliferation, modulate cellular differentiation, promote the production of extracellular matrix and angiogenesis, reduce inflammation, and speed up the healing process [27,28,29,30,31,32].

In the last two decades, the topical use of autologous platelet concentrates (PCs), such as platelet rich-plasma (PRP), has gained great popularity in a variety of human medical fields; for example, in dentistry [33,34,35], orthopedics, ophthalmology [36,37,38,39,40,41,42], wound healing [43,44,45,46], dermatology, and cosmetic and plastic surgery [47,48,49] for its regenerative and anti-inflammatory properties.

Despite the worldwide use of PRP, there are no standardized and universally shared protocols in PRP production, especially for determining the presence and number of leukocytes in the product [50]. The presence of a detectable content of leukocytes in an injectable preparation of PRP, known as leucocyte and platelet-rich plasma (L-PRP), can increase the in situ production of growth factors, with antibacterial activity and potential analgesic effect [51,52,53], and the release of high levels of pro-inflammatory cytokines, such as TNF-α and IL-1β, which increase the catabolism of the extracellular matrix [54,55].

PCs have antibacterial properties, as evidenced by in vitro and in vivo studies conducted mostly in humans using *Staphylococcus aureus*, *Staphylococcus epidermidis*, and *Escherichia coli* [56,57,58,59] and also in laboratory animals, such as rabbits [60]. Some studies with PCs have been conducted against methicillin-sensitive and methicillin-resistant *S. aureus* (MRSA) in horses with interesting results [61,62].

Despite continued and growing interest, fewer in vitro [63,64] and in vivo studies on the promising regenerative role of PCs have been conducted in dogs [65,66,67,68,69,70], and only two recent studies have explored the potential association between PCs and bacterial growth. In a small controlled clinical trial, methicillin-resistant *S. aureus* (MRSA) experimentally infected canine skin wounds treated with PRP demonstrated an increased healing process with a diminution of inflammation and bacterial loads [27]. A more recent in vitro study highlighted the antibacterial effect of some non-transfusional hemo-components in dogs against various susceptible, multidrug- and pan-drug-resistant bacteria, such as *Staphylococcus aureus*, *Escherichia. coli*, *Pseudomonas aeruginosa*, and *Klebsiella pneumoniae* [71].

To the authors’ knowledge, no study has yet been carried out to evaluate the antibacterial effect of canine PCs on *S. pseudintermedius*. Therefore, this in vitro study aimed to evaluate the antimicrobial effect of canine L-PRP and platelet-poor plasma (PPP) against two field strains of *S. pseudintermedius* isolated from dogs affected by pyoderma: one MDR strain and one non-MDR strain. A further aim was to evaluate the effect of activation on the antimicrobial potential of L-PRP. Our hypothesis was that L-PRP, with its larger platelet count and therefore increased concentration of growth factors, would show superior antimicrobial efficacy on both bacterial strains compared to PPP and that in vitro activation would increase the L-PRP antimicrobial effect.

## 2. Materials and Methods

### 2.1. Animals and Blood Collection

Twenty healthy, un-sedated, adult blood donor Golden Retriever dogs (PLT count within canine reference range), 12 males and 8 females, with ages between 1.5 and 10 years (4.63 ± 2.75 years) accepted to the Veterinary Transfusion Research Laboratory (REVLab) of the Department of Veterinary Medicine and Animal Science, University of Milan, for routine blood donor check-ups were enrolled in this study. All dogs were fasted for 12 h before blood sample collection. The study was carried out with client-owned dogs after approval by the University of Milan Animal Welfare Bioethical Committee (Approval number OPBA_101_2018) and with informed owner consent.

### 2.2. Preparation of L-PRP and PPP

Whole blood (WB) was collected and L-PRP and PPP were produced according to the manufacturer’s instructions with a closed semi-automatic system for veterinary use (CpunT 20, Eltek group, Casale Monferrato, Alessandria, Italy) previously used in dogs [64,70].

The system comprises a single-use, sterile blood collection kit (CpunT 20 mL, Eltek S.p.A., Hone, AO, Italy), a dedicated centrifuge (Eltek Group, Casale Monferrato, Alessandria, Italy), and an automated device for the separation of the L-PRP (Eltek Group, Casale Monferrato, Alessandria, Italy). The collection equipment comprised a butterfly needle (19G) attached to a 20 mL syringe for blood aspiration, an antibacterial filter on the access port for the addition of an anticoagulant (3 mL of 3.8% sodium citrate, PKL-Paramedical s.r.l, Salerno, Italy), and a 10 mL bag for the storage of L-PRP. Twenty mL of whole blood (WB) was collected aseptically from the cephalic vein of each dog and then mixed with 3 mL of anticoagulant previously loaded into the collection kit.

After sample collection, only the aspiration syringe united to the 10 mL storage bag was centrifuged at 1200× *g* for 15 min in the special centrifuge. At the end of the centrifugation erythrocytes, buffy coat, and supernatant plasma layers were visible in the aspiration syringe which was placed in the automated device for the separation. The movement of a vertical plunger directed by an optical reader isolated the supernatant plasma, the buffy coat, and the surface of the erythrocyte layer into the storage bag. The aspiration syringe with only the red blood cell layer inside was then separated from the bag. Next, the storage bag was centrifuged again at 2000× *g* for 5 min to separate the platelet pellet from the surrounding platelet-poor plasma (PPP). Finally, using a sterile syringe throughout the appropriate perforable membrane, 75% of the supernatant PPP was removed and collected in an Eppendorf for the following step.

The pellet was resuspended in 25% of the residual PPP by soft manual agitation of the storage bag to produce the leukocyte- and platelet-rich plasma (L-PRP).

For each dog, leucocyte count (WBC/µL) and platelet count (PLT/µL) in an aliquot of WB and L-PRP were calculated by an automatic analyzer (Cell-Dyn 3500 analyzer, Abbott Diagnostics Europe, Wiesbaden, Germany). The increment in platelet concentration in L-PRP over whole blood baseline values was determined using the following equation: platelet count L-PRP/platelet count WB. All samples were stored at room temperature on a laboratory blood roller mixer for 5 min before counts were performed. L-PRP and PPP were used immediately after production.

### 2.3. L-PRP Activation

A 1 mL aliquot of 10 L-PRP randomly selected samples was taken from the storage bag and put in a dedicated Eppendorf and activated with the addition of 100 μL of a bovine thrombin solution (500 IU/mL, BioPharm Laboratories LLC, Bluffdale, UT, USA), as previously described [72]. After activation, the samples were kept at 37 °C in an incubator. After three hours, the supernatant (after spontaneous clot retraction) from each L-PRP was collected and used for the next step.

### 2.4. Bacterial Strains: Identification, Antimicrobial Resistance PROFILE Determination, and Culture Conditions

Two SP field strains (SP40 and SP67) derived from previous microbiological examination (both SP strains had been isolated from clinical cases of canine pyoderma), were profiled, and categorized according to their antimicrobial resistance profile. As previously reported, the species was determined using phenotypic and molecular approaches [7,73]. Briefly, after isolation on Trypticase Soy Agar (TSA) +5% defibrinated sheep blood agar (Microbiol, Uta, Sardinia, CA, Italy) and Mannitol Salt Agar (Microbiol, Uta, Sardinia, CA, Italy), staphylococcal colonies were confirmed by standard phenotypic techniques (e.g., Gram stain, catalase test, and coagulase test). Following DNA extraction by boiling method [74], species-specific primer pairs were used to selectively amplify a fragment of the thermonuclease (*nuc*) gene [75].

The antibiotic resistance profile was determined by the Kirby–Bauer disc diffusion method according to the Clinical Laboratory and Standards Institute guidelines, and the panel of antibiotics used was the same as previously reported [7]. SP40 was classified as non MDR while SP67 was considered MDR. Moreover, the literature search revealed two multiplex PCR (M-PCR1 [76] and M-PCR 2 [77]) reactions and these were used to amplify antibiotic resistance genes (ARGs). M-PCR1 targeted *mecA* and *blaZ* genes, while M-PCR 2 *tetK*, *tetM*, and *aacA-aphD* genes, nucleotide sequences, and amplification conditions were as already described [76,77]. Table 1 reports the antimicrobial resistance profiles of microorganisms used in this study.

To evaluate the antimicrobial activity of L-PRP, glycerol stock solutions of SP40 and SP67 stored at −20 °C were thawed at room temperature prior to spreading 10 µL on TSA +5% defibrinated sheep blood agar and incubated for 24 h at 37 °C. Three to five bacterial colonies were collected and dispersed in Mueller–Hinton broth (MH, Microbiol, Uta, Sardinia, CA, Italy) to reach a final concentration equivalent to 0.5 McFarland Standard (containing approximately 1.5 × 10^8^ colony-forming-units (CFU)/mL). Thirty-three µL of those bacterial suspensions were added to each L-PRP and PPP tube and used for the next steps.

### 2.5. Antibacterial In Vitro Evaluation of L-PRP and PPP Antibacterial Activity

After production of L-PRP and PPP, their antimicrobial effect was evaluated by exposing SP strains to 10 L-PRP, 10 activated L-PRP, and 10 PPP samples, respectively. L-PRP, and activated L-PRP and PPP were mixed with the bacterial suspension in MH broth, and solutions were incubated at 37 ± 2 °C. Samples for bacterial growth determination were withdrawn from solutions at four timepoints: immediately after L-PRP, at activated L-PRP and PPP incubation (T0), at one hour (T1), and at two hours (T2) of incubation. Samples were serially diluted 1:10 in distilled water and 10 µL from each serial dilution was plated on TSA +5% defibrinated sheep blood agar and incubated for 24 h at 37 °C. After incubation, the number of CFUs in each plate was manually determined. The positive control consisted of bacteria grown in Mueller–Hinton broth to generate a standard curve. The negative control was Mueller–Hinton broth alone. The experiments, including CFU counts, were performed in triplicate to derive mean and standard deviation (SD) [52].

## 3. Statistical Analysis

Results are presented as mean ± standard deviation. The normal distribution of data were assessed using the D’Agostino and Pearson normality test and a non-normal distribution was confirmed. The antimicrobial effect of L-PRP, and activated L-PRP and PPP on two SP strains (SP40 and SP67) compared to positive control, to each other, and between the three timepoints was evaluated with the Wilcoxon test. For statistical analysis, we divided the sample by sex (male vs. female) and age (possible range of donors aged between 2 and 8 years [10]: ≤4 years—young adult vs. >5 years—adult), and we evaluated the influence of these variables on L-PRP and PPP antimicrobial effects with the Mann–Whitney test. We also divided the L-PRP samples by platelet count (≤700.000 platelet/µL vs. >700.000 platelet/µL), and we evaluated the influence of this variable on the L-PRP antimicrobial effect with the Mann–Whitney test. A *p* value of <0.05 was accepted as statistically significant. Statistical analyses were performed using commercial software (MedCalc^®^ Statistical Software version 20.027, MedCalc Software Ltd., Ostend, Belgium). Graphic representations were performed on Medcalc and GraphPad Prism version 8.0.0 for Windows (GraphPad 10 Software, San Diego, CA, USA).

## 4. Results

### 4.1. L-PRP and PPP Values and Overall Antimicrobial Effects

No technical problems occurred during L-PRP and PPP preparation and L-PRP activation. The mean platelet count and the mean leukocyte count for all twenty L-PRP samples (before activation) were 733,750 ± 167,411 PLT/µL and 16,210 ± 3368 WBC/µL, respectively. In L-PRP, platelet concentrations increased 4-fold compared with the whole blood (WB) baseline value. The highest PLT concentration was 1,217,000/μL, the lowest value was 416,000/μL. The mean volume of L-PRP obtained was 2.1 ± 0.8 mL. The mean platelet count in PPP was 33,000 ± 14,399 PLT/µL.

The positive control had a statistically higher mean CFU/mL count in comparison to both L-PRP and PPP for both bacterial strains (SP40 and SP 67) and for all three timepoints, with *p* = 0.005 for the comparison L-PPP and positive control at T0, and *p* = 0.002 for all the other comparisons (Table 2).

### 4.2. Evaluation of Antimicrobial Effect of L-PRP

**Figure 1:**
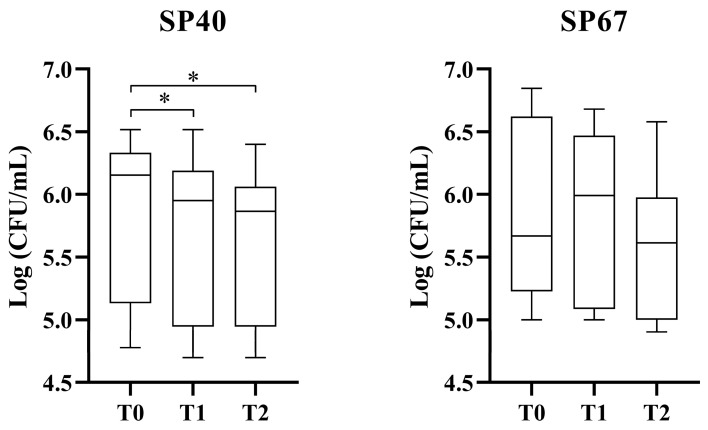
Antibacterial effect of L-PRP against non-MDR and MDR SP strains. * statistically significant.

Figure 1 reports the antibacterial activity of L-PRP against SP40 and SP67. Results demonstrated a bacteriostatic effect of this hemo-component with a statistically significant reduction in bacterial growth for SP40 (non-MDR strain) at one and two hours of incubation (*p* < 0.001 between both T0–T1 and T0–T2). The MDR strain (SP67) showed a progressive, but not statistically significant, decline in growth during the three timepoints (*p* = 0.37, 0.06, and 0.16, respectively).

### 4.3. Evaluation of Antimicrobial Effect of PPP

**Figure 2:**
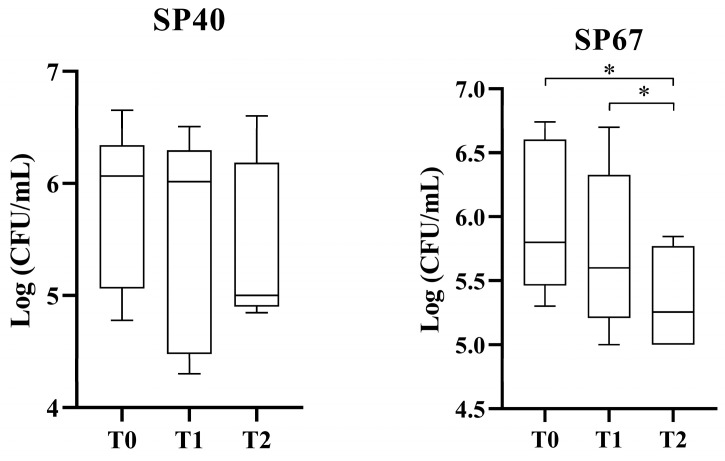
Antibacterial effect of PPP against non-MDR and MDR SP strains. * statistically significant.

There was no statistically significant difference in the PPP antimicrobial effect against the non-MDR strain (SP40) at the three timepoints (*p* = 0.08, 1.0 and 0.28) There was a statistically significant increase in the antimicrobial effect against the MDR strain (SP67) between T0–T2 and T1–T2 (*p* = 0.01 and *p* = 0.004, respectively) (Figure 2).

### 4.4. Comparison Between Antimicrobial Effect of L-PRP and PPP

Contrary to expectations, there were no statistically significant differences between the L-PRP and the PPP concerning the antimicrobial effect against the two SP strains tested (Table 2 and Supplementary Material).

### 4.5. Evaluation of Variables That Can Influence Antimicrobial Effect of L-PRP and PPP

**Figure 3:**
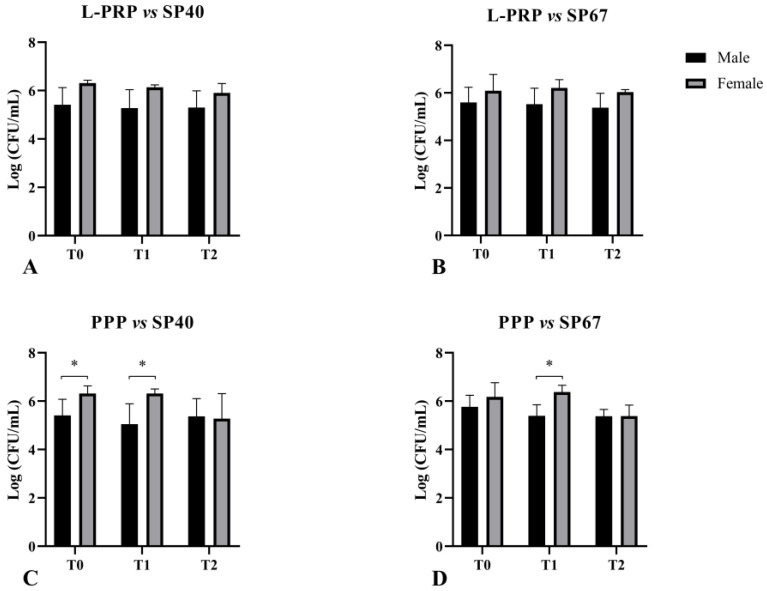
Effect of animal sex on the antibacterial activity of L-PRP (**A**,**B**) and PPP (**C**,**D**) against SP strains. * statistically significant.

PPP showed a statistically higher antimicrobial effect in male dogs against the nonMDR strain at T0 (*p* = 0.04) and at T1 (*p* = 0.02) and against the MDR strain at T1 (*p* = 0.02) (Figure 3). No other association (age or platelet count in L-PRP) was statistically significant.

### 4.6. Evaluation of Antimicrobial Effect of Activated L-PRP

The positive control had a statistically higher CFU/mL count in comparison with activated L- PRP for both bacterial strains (SP40 and SP 67) and for all three timepoints with *p* = 0.002, except for T1 for SP40 (*p* = 0.19) (Table 2)

**Figure 4:**
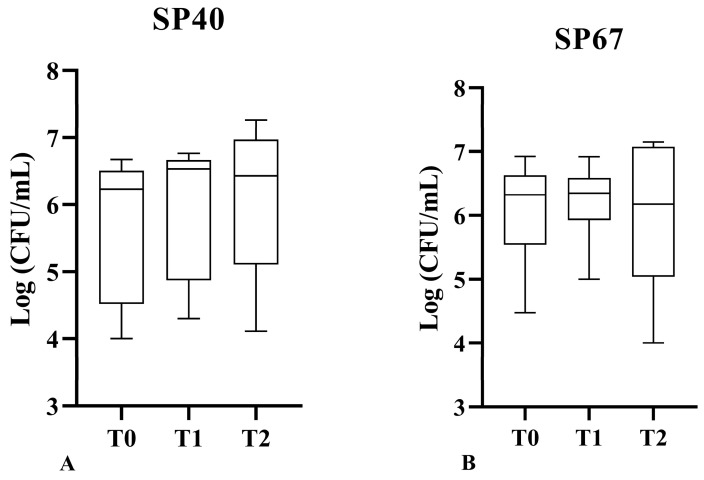
Activated L- PRP antibacterial action over time for SP40 (**A**) and SP67 (**B**) strains.

There were no statistically significant differences for the antimicrobial effect of activated L-PRP against non-MDR and MDR strains (SP40 and SP67) at the three timepoints (*p* = 0.13, 0.06, and 0.30 and *p* = 0.82, 0.57, and 0.95, respectively), with maximum bacteriostatic activity at the time of incubation (T0) (Figure 4).

Finally, activated L-PRP had a similar antimicrobial effect against the non-MDR and MDR strains compared to L-PRP at all three timepoints, with no statistically significant differences (*p* = 0.82, 0.13, and 0.08 and *p* = 0.62, 0.30, and 0.23, respectively).

## 5. Discussion

This in vitro study is the second to be carried out on canine L-PRP, and the first to use two field strains of *S. pseudintermedius*, the main pathogen implicated in canine superficial pyoderma. The possible clinical implications are therefore of considerable importance, especially considering the growing antibiotic resistance evident in canine skin infections caused by this bacterium [6,10,20,21]. In this study, we chose to use only two *S. pseudintermedius* field strains, one MDR and one non MDR, so that we focus on evaluating the possible diversity of L-PRP and PPP activities from donors with different characteristics. Our results suggest that both canine L-PRP and PPP have an in vitro statistically significant, if not even dramatic, bacteriostatic effect against these non-MDR and MDR *S. pseudintermedius* strains, which exceeds two hours, since the greatest inhibitory efficacy for both products in both strains occurred at T2. These data are consistent with all recent systematic reviews in human medicine focused on in vitro, preclinical, and clinical investigations of the antibacterial potential of PCs [78,79,80], and with veterinary studies achieved in vitro concerning the possible antimicrobial effects of animal hemo-components on many bacteria other than *S. pseudintermedius* [61,62,71,81,82,83]. Among these, only one study was conducted in dogs [71], and it reported the antibacterial properties of different canine hemo-components for non-transfusional use (PRP, L-PRP, platelet gel, platelet lysate, fibrin glue, PPP) and of activating substances (thrombin, calcium gluconate) against Gram-positive (*S. aureus* subsp. *aureus*, *S. cohnii* subsp. *cohnii*) and Gram-negative bacteria (*P. aeruginosa*, *E. coli*, *K. pneumoniae* subsp. *pneumoniae*) isolated from canine wounds and categorized as susceptible, resistant, or MDR to a panel of known human and veterinary antibiotics. Some in vivo experimental studies using animal models have also proved the antibacterial effect of non-transfusional blood components: PRP displayed antimicrobial properties in rabbit with osteomyelitis [83,84], enhanced healing of experimentally infected surgical wounds in rats [85], and increased healing of spontaneous or experimentally generated infected canine skin wounds by demonstrating antibacterial activity, prompt inflammation reduction, quick granulation tissue formation, and re-epithelialization [27,66,69].

L-PRP resulted in a statistically significant reduction in bacterial growth for the non-MDR SP strain at all three timepoints, whilst in the MDR SP strain there was only a trend for an increase in inhibition during the timepoints, which was not statistically significant. These data agree with the equine literature on *S. aureus*, which suggests that MSSA is more sensitive than MRSA to PRP treatment [61]. PPP showed an opposite trend compared to L-PRP, since it demonstrated a constant, but not statistically significant, increasing efficacy against the non-MDR SP strain at the three timepoints, while against the MDR SP strain its inhibitory efficacy increased in a statistically significant manner during the three timepoints, particularly at T2. These data are not supported by the literature. In fact, in horses, PPP, like PRP, tends to be more effective against MSSA than against MRSA [61]. Therefore, in our study, PPP, although unusually employed in regenerative medicine for therapeutic purposes, appeared to have antimicrobial potential similar to PRP against *S. pseudintermedius*. These results suggest, as in human medicine, that the antimicrobial bacteriostatic action of platelet derivates, especially against the MDR strain, might be related to plasma components, such as complement, rather than the platelets [53,86].

The results from this study suggest that canine-activated L-PRP also has an apparent bacteriostatic effect against both non-MDR and MDR *S. pseudintermedius* field strains when compared to positive control. However, this is apparently less long-lasting than L-PRP since the maximum level of inhibition was exerted at T0, and then its efficacy decreased at subsequent timepoints in both bacterial strains tested. This difference compared to non-activated L-PRP might be explained by the presence of the bovine thrombin used for activation in this study, which may influence the inhibition properties of L-PRP as shown in horses [81]. This is in contrast with what was reported in a previous study regarding the antibacterial efficacy of platelet derivatives against *S. aureus* in humans in which no inhibitory effect was observed using 12 µL of thrombin [87] and in a veterinary study on Gram-negative bacteria in dogs [71], in which bovine thrombin instead seemed to enhance the bacteriostatic activity of PCs only against Gram-negative bacteria. In both of these studies, however, it was reported that the effect of thrombin depends greatly on the bacterium tested and maybe the source (autologous or bovine thrombin); therefore, the results from these bacteria may not be extrapolated to *S. pseudintermedius*, and further studies are needed to clarify the mechanism of action.

In our study, L-PRP from male dogs seemed to have greater antimicrobial effects in both bacterial strains tested, but no correlation was found between L-PRP antimicrobial effects and donor age or L-PRP platelet concentration. While the result regarding the male sex was unexpected and is not reported in the literature, the result regarding platelet concentration is in line with the literature, since it has already been shown in human medicine that the antibacterial activity of PRP is not correlated to the platelet numbers [87,88]. Regarding the influence of donor age on L-PRP antibacterial effect, in humans it has been demonstrated that PRP from young subjects has a greater in vitro regenerative effect [89,90], but no study has been published regarding the donor age and antibacterial PRP activity correlation either in human or veterinary medicine.

The average platelet concentration in our L-PRP was 733.750 ± 167.411 PLT/µL, similar to that obtained by other authors [64,71] and increased 4-fold compared with the whole blood baseline value. According to the literature, this defines the concentrate obtained as a therapeutic PRP suitable for clinical use [32]. Some authors hypothesize that the cell counts are important to delineate the standard of hemo-components preparing procedures, linking the platelet concentration to the clinical regenerative outcome, as it is positively associated with growth factor concentration [91], but, as stated before, not correlated to antibacterial potential.

Our study has some limitations: we did not include PRP without leukocytes, but Attili’s study on dogs [71] demonstrated that the presence of leukocytes does not appear to be significant in determining the antimicrobial effect of blood components, as already hypothesized by previous studies [92,93]. Only a small number of dogs were used in this study, but using L-PRP produced by different dogs and individually tested instead of pooled blood, as conducted in others studies [71], more closely simulates a clinical setting. This, however, also increased the variability of the results obtained. In our study, we only evaluated the colonies for two hours after incubation and this did not allow us to evaluate the effect of the tested blood components over the long term. This choice was dictated by the modest quantity of products available for each subject and by the fact that previous studies regarding the timing of the bacteriostatic effect of platelet derivates in horses have shown that the broth method has a greater effect in the early hours [62].

Other limitations are that we did not test the effect of bovine thrombin alone on bacterial growth, we did not test more *S. pseudintermedius* strains, or we did not directly compare activated and non-activated L-PRP on the same subject, which may help to better define the role of L-PRP activation in the inhibiting bacterial growth. These limitations were mainly dictated by the small volume of L-PRP available for each subject (around 2 mL) which allowed for only a limited number of tests. Finally, in evaluating L-PRP, we did not measure the growth factors, as was conducted in studies on the horse [61,62]. However, a previous study using the same methodology demonstrated an adequate content of growth factors of L-PRP in dogs obtained with this method [64].

Although the antimicrobial effect of L-PRP and PPP detected by this study, even if statistically significant, was not as powerful as expected, our very promising results are focused on clinical SP strains and, in particular, on a MRSP isolate; thus, providing information on the interaction with a high virulent strain should stimulate further studies to confirm and understand the real mechanism, not yet completely understood, of the antibacterial effect of canine platelet derivates on *S. pseudintermedius* strains.

Due to the complexity of canine plasma, the fact that the chemical constituents of L-PRP and PPP were not monitored by the authors and that it is complex to define a target of antibacterial effect to be achieved in vitro that can have a real clinical effect, however the results of this study cannot be generalized or transposed in vivo, but the rise in MDR and pan-drug-resistant bacteria, creating an important health and economic risk for humans and animals, provides a valid impetus to investigate in this direction.

## 6. Conclusions

As previously described for other bacterial species, both L-PRP and PPP have an in vitro antimicrobial effect against the field strains of *S. pseudintermedius*, and the bacteriostatic effects of L-PRP and PPP are similar against both non-MDR and MDR strains. Activation seems to reduce the duration of the antimicrobial effect of L-PRP. This is the first study using field strains of *S. pseudintermedius*, and further studies are needed to confirm and strengthen these results. Moreover, some intrinsic variables (e.g., sex) seem to influence the antimicrobial effect of platelet derivates. Our conclusion, however, cannot be generalized to all *S. pseudintermedius* strains, and more studies are necessary to confirm these preliminary results.

## Figures and Tables

**Table 1:** Antimicrobial resistance profiles of SP strains used in this study.

Bacterial ID	Phenotypic Resistance Profile	Molecular Resistance Profile	Genome Length (bp)
SP40	AML	*blaZ*	2528276
SP62	OX, AMC, CL, CVN, EFT, DA, DO, ENR, MAR, P, CRO, MY, AML, CAR, AZM, E, K	*mecA*, *tetK*, *blaZ*, *aacA-aphD*	2895334

Abbreviations: OX = oxacillin, AMC = amoxicillin + clavulanate, CL = cephalexin, CVN = cefovecin, EFT = ceftiofur, DA = clindamycin, DO = doxycyline, ENR = enrofloxacin, MAR = marbofloxacin, P = pradofloxacin, CRO = ceftriaxone, MY = lincomycin + spectinomycin, AML = amoxicillin, CAR = carbenicillin, AZM = azithromycin, E = erythromycin, and K = kanamycin.

**Table 2:** Antibacterial effect of L-PRP and PPP against non-MDR and MDR SP strains compared to positive control (pos ctr) and negative control (neg ctr) during time. * statistically significant for timepoints comparison. All values of L-PRP, PPP, and activated L-PRP were statistically significant compared to positive control, except for activated L-PRP at T1, marked with ^§^. *p* Value is for comparison of the antibacterial activity between L-PRP and PPP against non-MDR and MDR SP strains.

Timepoint	Mean Log (Colony-Forming Units (CFU)/mL (1:1000 Dilution)					
	NON-MDR STRAIN (SP 40)					
	L-PRP	PPP	Activated L-PRP	Pos ctr	Neg ctr	*p* Value
T0	6.1	6.1	6.2	6.5	0	*p* = 1.0
T1	6 *	6.1	6.4 ^§^	6.5	0	*p* = 0.56
T2	5.9 *	5.9	6.7	7.3	0	*p* = 0.73
	MDR STRAIN (SP 67)					
T0	6.3	6.2	6.2	7	0	*p* = 0.38
T1	6.2	6.1 *	6.3	7	0	*p* = 0.91
T2	5.9	5.5 *	6.4	7.3	0	*p* = 0.06

## Data Availability

The data presented in this study are available upon request from the corresponding authors.

## Supplementary Materials

The following supporting information can be downloaded at: https://www.mdpi.com/article/10.3390/vetsci11120670/s1, Figure S1: Comparison of the bacterial activity of L-PRP and PPP.
