# Supplementary material for: Antibacterial Effect of Canine Leucocyte Platelet-Rich Plasma (L-PRP) and Canine Platelet-Poor Plasma (PPP) Against Methicillin-Sensitive and Methicillin-Resistant Staphylococcus pseudintermedius
Source: Vet Sci. 2024 Dec 20;11(12):670. doi: 10.3390/vetsci11120670 (PMC11680258; doi:10.3390/vetsci11120670)
Supplement: Supplementary file 1 [file vetsci-11-00670-s001.zip › vetsci-3301868-supplementary.pdf]

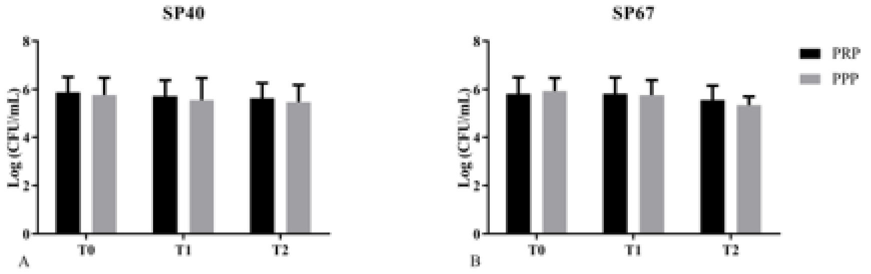

Figure S1. Comparison of the antibacterial activity of L-PRP and PPP against non MDR (A) and MDR (B) SP strains.
